# People With Parkinson’s Disease and Freezing of Gait Show Abnormal Low Frequency Activity of Antagonistic Leg Muscles

**DOI:** 10.3389/fnhum.2021.733067

**Published:** 2022-01-26

**Authors:** Maria-Sophie Breu, Marlieke Schneider, Johannes Klemt, Idil Cebi, Alireza Gharabaghi, Daniel Weiss

**Affiliations:** ^1^Centre of Neurology, Department of Neurodegenerative Diseases, University of Tübingen, Tübingen, Germany; ^2^Hertie Institute for Clinical Brain Research, Tübingen, Germany; ^3^Centre for Neurosurgery, Institute for Neuromodulation and Neurotechnology, University of Tübingen, Tübingen, Germany

**Keywords:** Parkinson’s disease, EMG, freezing of gait (FOG), low frequency activation, DBS (deep brain stimulation)

## Abstract

**Objective:**

Freezing of gait is detrimental to patients with idiopathic Parkinson’s disease (PD). Its pathophysiology represents a multilevel failure of motor processing in the cortical, subcortical, and brainstem circuits, ultimately resulting in ineffective motor output of the spinal pattern generator. Electrophysiological studies pointed to abnormalities of oscillatory activity in freezers that covered a broad frequency range including the theta, alpha, and beta bands. We explored muscular frequency domain activity with respect to freezing, and used deep brain stimulation to modulate these rhythms thereby evaluating the supraspinal contributions to spinal motor neuron activity.

**Methods:**

We analyzed 9 PD freezers and 16 healthy controls (HC). We studied the patients after overnight withdrawal of dopaminergic medication with stimulation off, stimulation of the subthalamic nucleus (STN-DBS_only)_ or the substantia nigra pars reticulate (SNr-DBS_only_), respectively. Patients performed a walking paradigm passing a narrow obstacle. We analyzed the frequency-domain spectra of the tibialis anterior (TA) and gastrocnemius (GA) muscles in ‘regular gait’ and during the ‘freezing’ episodes.

**Results:**

In stimulation off, PD freezers showed increased muscle activity of the alpha and low-beta band compared to HC in both TA and GA. This activity increase was present during straight walking and during the freezes to similar extent. STN- but not SNr-DBS decreased this activity and paralleled the clinical improvement of freezing.

**Conclusion:**

We found increased muscle activation of the alpha and lower beta band in PD freezers compared to HC, and this was attenuated with STN-DBS. Future studies may use combined recordings of local field potentials, electroencephalography (EEG), and electromyography (EMG) to interrogate the supraspinal circuit mechanisms of the pathological activation pattern of the spinal pattern generator.

## Introduction

Freezing of gait (FoG) in Parkinson’s disease (PD) represents the defective spinal motor output depending on the supraspinal cortical, subcortical, and brainstem contributions (Lewis and Shine, 2016; Snijders et al., 2016; Weiss et al., 2020). As such the motor, cognitive, and emotional systems (Shine et al., 2013; Ehgoetz Martens et al., 2018) modulate the effective spinal motor output according to the environmental and internal requirements (Drew et al., 2004; Snijders et al., 2016). In particular, the spinal pattern generator is modulated by the descending drives of the pyramidal tract and the nigro-ponto-reticulospinal pathway (Nutt et al., 2011; Snijders et al., 2016).

The rhythmic alternation of stepping during regular gait is generated in the spinal cord in humans (Guertin, 2009). Historically, early experimental studies in decerebrate cats suggested that the spinal pattern generator generates rhythmic locomotion, even in the absence of supraspinal input. The cats were able to walk, trot and gallop when put on a treadmill, but the gait was mechanical and inflexible (Mori, 1987; Snijders et al., 2016). Further, experimental models found that the descending drives to spinal motor neurons stem from nuclei of the mesencephalic locomotor region (MLR) including the pedunculopontine and cuneiform nucleus and the reticulospinal projections (Jordan et al., 2008; Takakusaki, 2013; Snijders et al., 2016). Previous experimental and clinical research supported that human gait can be modulated both on the level of the substatia nigra, pars resticulate (SNr) based on the monosynaptic GABAergic projection from the SNr (Weiss et al., 2013; Scholten et al., 2017; Heilbronn et al., 2019; Valldeoriola et al., 2019) to the pedunculo-pontine nucleus (PPN) (Ferraye et al., 2010; Garcia-Rill et al., 2019). Experimental research suggested that both dopaminergic depletion and pharmacological des-inhibition increased GABAergic SNr activity resulting in a pro-akinetic net effect (Burbaud et al., 1998; Breit et al., 2006). Instead, pharmacological or electrical SNr inhibition led to prokinetic effects including those on gait (Wichmann et al., 2001; Lafreniere-Roula et al., 2010; Sutton et al., 2013; Milosevic et al., 2018). Experimental research established a reciprocal link between SNr and PPN, showing the reciprocal regulation of single cell activity of the two nuclei (Breit et al., 2001, 2006). In human PD, nigral stimulation modulated clinical and kinematic gait measures (Scholten et al., 2017; Heilbronn et al., 2019) and FoG. Yet, it has to be kept in mind that these conclusions stem from piloting observations and have to be confirmed in larger clinical trials (Snijders et al., 2016; Garcia-Rill et al., 2019; Weiss et al., 2020).

Less so the model work but more the clinical and neurophysiological human PD gait research pointed to the fact that the subthalamo-cortical circuits may be more meaningful in PD gait than was anticipated in experimental work. In particular, patients with L-Dopa sensitive FoG may show considerable therapeutic benefit from subthalamic nucleus deep brain stimulation (STN-DBS) (Schlenstedt et al., 2017; Barbe et al., 2020; Cebi et al., 2020). The STN holds both inhibitory indirect and hyperdirect projections to the primary motor cortex (Delwaide et al., 2000; Gradinaru et al., 2009), and high-frequency stimulation of the STN modulated activity and excitability of the primary motor cortex (Kuriakose et al., 2010; Udupa et al., 2016) as well as of the associated premotor and prefrontal cortical areas (de Hemptinne et al., 2015; Weiss et al., 2015). However, stimulation of the STN does not exclusively act on the subthalamo-cortical circuit, but also entrains brainstem connections including in PD gait (Pötter et al., 2008). The subthalamic contributions to PD gait and freezing phenomena have more recently been highlighted with neurophysiological techniques studying oscillatory activity of STN local field potentials and cortical activity. Traditionally, enhanced broad alpha and beta band activity (8–35 Hz) from STN-LFPs correlated with bradykinesia and rigidity (Brown, 2003; Kühn et al., 2006, 2009) and were suppressed with effective STN-DBS therapy (Kühn et al., 2008; Eusebio et al., 2012). More recent studies linked oscillatory activity with freezing phenomena. As such, PD freezers showed elevated activation around 18 Hz at movement initiation (Storzer et al., 2017) compared to non-freezers. Additionally, pathologically prolonged broad band beta burst duration (Tinkhauser et al., 2017) was associated to freezing, since beta burst duration (13–30 Hz band) was more prolonged in PD freezers compared to non-freezers during regular gait and more pronounced during the freezing episodes (Anidi et al., 2018).

In addition to STN, the cortex is involved in freezing phenomena as indicated from oscillatory activity and cortical stimulation studies, as reviewed elsewhere (Weiss et al., 2019). Briefly, upper limb freezing (ULF) showed enhanced activity around and below 10 Hz during a freeze in the alpha band (Scholten et al., 2016a). Moreover, cortical abnormalities of both cortico-cortical synchronization (Scholten et al., 2016b) and beta band decoupling abnormalities prior to a freeze indicated premonitory cortical susceptibility to freezing (Scholten et al., 2020). There were similar findings in FoG, when cortico-subthalamic decoupling in the low frequency band (4–13 Hz) became evident not only during freezing episodes, but also preceded a freeze (Pozzi et al., 2019). Finally, similar abnormalities were found in the PPN when freezing episodes showed attenuated alpha activity (Thevathasan et al., 2012), and electromyography (EMG) studies showed enhanced activity below 10 Hz in the PD off state in general (Salenius et al., 2002; Weiss et al., 2012). More specific, activity around and below 10 Hz was found in freezers and during ULF, and was suppressed by STN-DBS (Scholten et al., 2016a).

Little is known about the pathological changes in the frequency domain in muscular activity in PD patients when exhibiting FoG. Based on the current literature, we explored muscular activity in broad frequency range from 1 to 45 Hz comprising the above mentioned frequency bands, and compared to healthy controls (HC). Derived from these findings, we further explored if activation abnormalities of the alpha and beta frequency were related to freezing episodes. Then, we used DBS therapy to differentially modulate the basal ganglia contributions to the spinal motor neurons, applying both STN and SNr stimulation, respectively. We posited that – if the supraspinal contribution of each nucleus was relevant to spinal motor activity and FoG – neuromodulation of either target should modulate both the clinical expression of freezing and the activity of the antagonistic tibialis anterior (TA) and gastrocnemius (GA) muscles.

## Materials and Methods

### Subject Characteristics

We included 16 patients with idiopathic PD and DBS and 16 age- and gender-matched HC. From these, we selected 11 PD patients with clinically confirmed FoG episodes (Snijders et al., 2012). We excluded two further PD patients from analysis, one owing to technical problems during the recording and another owing to the inability to walk during the experimental session. Finally, we analyzed data from 9 PD freezers (3 female, age 66.4 ± 7.2 years) and 16 HC (6 female, age: 58.5 ± 4.6 years). Detailed patient characteristics are given in Table 1.

**TABLE 1:** Patient characteristics of the final analysis cohort; NFOG-Q, New Freezing of Gait Questionnaire (Seuthe et al., 2021); STN-DBS, deep brain stimulation of the subthalamic nucleus; SNr-DBS, deep brain stimulation of the substantia nigra pars reticulata.

ID	Gender	Age	Disease duration (years)	Months with DBS	Disease dominant side (L/R)	NFOG-Q	Motor score (UPDRS III, item 18–31) OFF/STN/SNr	MMST	STN-DBS parameters				SNr-DBS parameters			
									Voltage (left/ right)	Frequency (Hz)	pulse width (μs)	Active contacts (left/ right)	Voltage (left/ right)	Frequency (Hz)	Pulse width (μs)	Active contacts (left/ right)
2	M	69	17	13	L	13	49/49/49	27	2.7/3.0	130	60	2–3+/ 10–11+	2.5/2.5	130	60	0–1+/ 8–9+
4	M	64	16	86	L	4	73/40/61	30	5.3/3.0	125	60	2–3+/ 6–7+	3.5/3.5	125	60	0–1+/ 4–5+
5	M	71	16	27	R	4	50/28/x	30	3.6/3.6	130	60	2–3+/ 10–11+	x/x	x	x	x
8	M	64	9	14	R	10	50/28/32	27	2.8/3.5	130	60	2–3+/ 10–11+	2.5/1.9	130	60	0–1+/ 8–9+
9	F	56	21	61	R	4	54/31/40	30	5.5/3.5	130	60	2–3+/ 6–7+	2.9/2.9	130	60	0–1+/ 4–5+
10	M	55	17	4	R	15	48/28/31	28	4/4.5	130	60	2–3+/ 10–11+	2.7/2.7	130	60	0–1+/ 8–9+
13	F	76	13	6	L	6	46/34/41	27	2.1/2.1	125	60	2–3+/ 10–11+	1.6/1.6	125	60	0–1+/ 8–9+
14	F	76	19	10	R	12	38/25/35	28	3.2/2.0	130	60	2–3+/ 10–11+	2.2/2.2	130	60	0–1+/ 8–9+
15	M	67	16	11	R	7	62/28/67	29	5.4/5.1	130	60	2–3+/ 10–11+	1.3/1.3	130	60	0–1+/ 8–9+

*In PD 5 electrode contacts did not reach SNr.*

Inclusion criteria were idiopathic PD with akinesia-rigidity type and time since DBS implantation more than 3 months. Exclusion criteria were Mini Mental Status Examination < 22, Beck’s Depression Inventory > 13, and other neurological or neuromuscular disease except PD. The local Ethics committee of the University of Tübingen approved the study (application no. 732/2012BO2) and all subjects provided written consent to participate in the study.

### Experimental Setup

#### Paradigm

During the experimental session, patients walked repeatedly on a straight over ground walkway of 9 m forth and back. We installed two obstacles at 1/3 and 2/3 along the hallway to narrow the pathway in order to provoke FoG episodes (Rahman et al., 2008). The patients self-initiated walking and walked in their self-selected, comfortable pace for about 3 min, or at least as long as possible, the minimum walking period analyzed in a single patient was 70 s. All patients walked freely except patient PD2, who wished to use a walking aid uniformly in all therapy conditions. Patients were studied in three stimulation conditions after overnight withdrawal of dopaminergic medication (MedOff). Therefore, we recorded patients in stimulation off (‘StimOff’), stimulation of only STN: STN_only_ (briefly ‘STN’), and stimulation of only SNr: SNr_only_ (briefly ‘SNr’), and the three conditions were delivered in randomized order. Electrode localization of the active contacts was located in the STN (electrode model 3389, Medtronic, Minneapolis, MN, United States), additionally in 8 out of 9 patients the lowermost electrode contact reached the SNr area {at least –5 mm below the midcommisural point [MCP, mean coordinates of the cohort: left SNr: –11.0 (±0.6), –3.6 (±0.4), –7.1 (±0.5), right SNr: 10.3 (±0.5), –3.6 (±0.4), –6.2 (±0.3); left STN: –13.0 (±0.6), –1.4 (±0.4), –3.3 (±0.4); right STN: 12.1 (±0.4), –1.2 (± 0.4), –2.2 (±0.4); (x,y,z)]}, verified by co-registration of the preoperative MRI and postoperative CT images (Brainlab, München, Germany). Patients and experimenters were not blinded, and each stimulation condition was active for at least 20 min prior to the recording in order to achieve sufficient efficacy and to limit carry-over effects (Cooper et al., 2013; Weiss et al., 2013). Since the recordings took place in MedOff, we did not consider longer periods.

#### Kinematic and Electrophysiological Recordings

During walking, we recorded the synchronized videotapes as well as the kinematic and EMG time series. Therefore, patients wore small, lightweight body-fixed kinematic sensors attached to the left and right ankles (about 20 mm above the malleolus), and to the lumbar spine (APDM, Portland, OR, United States). Data was sampled at 128 Hz and transferred to Matlab (Release R2015b, The Mathworks, Inc., Natick, MA, United States) for the *post hoc* offline analysis. Detailed analyses of the kinematic features and the methodological approach were published elsewhere (Scholten et al., 2017). Briefly, gait kinematics including step length were only analyzed during effective walking, excluding freezing episodes. The events were calculated using the acceleration in the anterior–posterior direction and the angular velocity in the sagittal plane. First we identified the midswing (MS) as peak value exceeding 50°/s in the sagittal plane of the gyroscope signal. Next we identified toe-off (TO) and heel-strike (HS) in the time interval 750 ms before and after MS. TO was defined as minimum anterior–posterior acceleration in the time interval before MS, and HS was defined as the minimum value of angular velocity in the sagittal plane before the maximum anterior–posterior acceleration in the time interval after MS. Using the gait events, we computed temporal and spatial gait outcome measures for each condition.

Furthermore, we recorded bipolar EMG with active surface electrodes (actiCAP active Electrodes, Brain Products, Gilching, Germany) of the bilateral TA and GA muscles. We decided to use active electrodes which enabled the digitization of time series at electrode level and, from there, wireless transmission to the electroencephalography (EEG) recorder (EMG was recorded with an EMG input box connected and synchronized to our EEG-recording system), which helped prevent to expose the time series to cable swinging that would arise during gait (MOVE and active electrodes system, Brain Products, Gilching, Germany). The electrophysiological data was sampled at 1 kHz. Electrodes of the TA were placed 1/3 below the tip of the fibula on an imaginary line connecting fibula and the medial malleolus, the electrodes of the GA were placed over the most prominent bulge of the inner head. We used an inter electrode distance of 20 mm in accordance to the SENIAM Guidelines (Hermens et al., 2000).

#### Clinical Assessments

All patients reported narrative scores on FoG (NFOG-Q) 1 day prior to the recording. Moreover, we assessed the motor score in UPDRS III in each therapy condition (see Table 1). We deduced the objective freezing-related clinical information from the videos and kinematic survey while walking, i.e., number of freezing episodes, absolute time of freezing and the time percentage frozen (absolute time of freezing throughout the walking task over absolute duration of the walking task, see Table 2, individual parameters see Table 3).

**TABLE 2:** Freezing characteristics in different therapeutic conditions.

Condition	No of patients	No of freezing episodes	Absolute time frozen (seconds)	Time percentage frozen (%)
Off	4	15	144	39
STN	1	2	23	31
SNr	3	9	172	23

*Time percentage frozen is given as median value.*

**TABLE 3:** Single patient description of freezing episodes during straight walking (excluding U-turns at the end of the walkway).

ID	No of FoG Stim Off	No of FoG STN	No of FoG SNr	Absolute Time Frozen (seconds) Stim Off	Absolute Time Frozen (seconds) STN	Absolute Time Frozen (seconds) SNr	Duration single freezing episode (seconds) Stim Off min/max	Duration single freezing episode (seconds) STN min/max	Duration single freezing episode (seconds) SNr min/max	Time percentage frozen (%) Stim Off	Time percentage frozen (%) STN	Time percentage frozen (%) SNr
2	3	2	3	33	23	123	3/17	5/18	7/94	76	31	86
5	1	0	0	3	0	0	3	0	0	37	0	0
8	4	0	5	21	0	38	5/6	0	4/10	21	0	23
13	7	0	1	87	0	11	2/42	0	11	40	0	17

### Analyses

#### Data Segmentation, Preprocessing, and Spectral Analysis

For data analysis we selected the time series while walking straight ahead, and rejected the turning episodes. Next, we segmented for time series related to either ‘regular gait’ or ‘freezing episodes.’ We verified FoG episodes from the video recordings according to the existing consensus definition that defines FoG as ‘a brief, episodic absence or marked reduction of forward progression of the feet despite the intention to walk’ (Nutt et al., 2011), including shuffling episodes as well as complete movement arrests. To entirely remove complete freezing episodes from the ‘regular gait’ time series, we rejected the clinically defined FoG episode and 1 s before the episode.

We filtered the EMG data with a band pass finite impulse response filter from 10 to 200 Hz, notch filtered for the 50 Hz line artifact, and full-wave rectified the EMG time series (Mima and Hallett, 1999).

EMG signals were first partitioned into disjoint segments. Each time segment had a duration of 2 s resulting in a frequency resolution of 0.5 Hz. Every 200 ms the power spectral density of the muscular activity was computed of the segment using the fast Fourier transform (FFT; Matlab fft.m function). The FFT returns for each frequency bin a complex number, from which we extract the amplitude by taking the magnitude squared of this number to obtain the power spectral density expressed in μV2/Hz. We then averaged over all segments. We report the relative power spectral density after normalizing the absolute values to the summed power from 1 to 45 Hz.

Frequency spectra were computed for TA and GA separately for the left and right leg for PD and HC, as well as for the disease dominant and the non-dominant side separately in PD. Since we did not find statistical differences between the two groups in both comparisons (cluster-based permutation test), we report the average of both legs in all analyses.

### Statistical Analysis

Descriptive are given as mean ± standard deviation, except for the time percentage frozen given as median value, due to a non-parametric data distribution.

In the first part of our analysis we aimed to compare frequency spectra from 1 to 45 Hz. We decided to explore a broad frequency range of interest based on the fact that (i) frequency domain analysis in ambulatory EMG has only sparsely been studied before, and (ii) in cortical and basal ganglia showed diverse abnormalities in this broad frequency range with regard to motor symptoms, freezing or cognitive processes including the theta, alpha, and also broad beta band (de Hemptinne et al., 2015; Tinkhauser et al., 2017; Anidi et al., 2018; Fischer et al., 2018; Hell et al., 2018; Pozzi et al., 2019). First we compared the frequency spectrum of PD freezers in ‘StimOff’ and during ‘regular gait’ with HC in TA and GA. Furthermore, we compared in PD freezers in ‘StimOff’ the frequency spectra (1–45 Hz) of ‘regular gait’ and ‘freezing’ in TA and GA. Then, we analyzed the effect of stimulation of either target (STN_only_, SNr_only_, respectively) on muscle activity, comparing the frequency domain spectra of PD freezers in ‘regular gait‘ ‘StimOff’ with ‘STN,’ and ‘StimOff’ with ‘SNr’ for TA and GA.

For statistical comparison of the frequency-domain spectra, we used a cluster-based permutation test as implemented in the Fieldtrip toolbox to address for multiple comparisons (Maris and Oostenveld, 2007). This test is based on the Monte Carlo permutation principle and identifies significant changes between conditions using clusters of adjacent frequencies. We performed 5000 randomizations and considered an adjusted two-sided alpha level of *p* < 0.05 significant.

#### Individual Alpha/Low Beta Peak

Based on the results from the spectral frequency-domain analysis we defined the individual alpha/low beta peak frequency for ‘regular gait’ as well as for ‘freezing’ episodes in ‘StimOff’ in PD freezers. We first identified the peak frequency for TA and GA separately in the frequency range of interest as identified in the cluster analyses. Then, we calculated the individual mean alpha/low-beta peak amplitude of the GA/TA peaks. We used the individual peak frequency from ‘freezing’ episodes in ‘StimOff’ to extract the individual alpha/low beta power to compare the differences between ‘regular gait’ and ‘freezing’ episodes in PD freezers in ‘StimOff.’ Additionally the individual alpha/low beta power at the individual peak frequency from ‘regular gait‘ in ‘StimOff’ was used for statistical comparison of ‘StimOff’ vs. ‘STN’, and ‘StimOff’ vs. ‘SNr.’ Data were non-parametric distributed and statistical differences were analyzed with a Wilcoxon signed ranks test.

#### Correlation Analyses

We correlated spectral measures (individual peak frequency) with clinical outcome parameters such as: FoG (NFOG-Q, UPDRS III item 14 (freezing episodes), number of freezing episodes during straight walking, percentage frozen during the complete walking paradigm. To evaluate the specificity of the observed associations with FoG, we performed control analysis by correlating the spectral measures of other PD motor symptoms including rigidity (UPDRS III item 22) and bradykinesia of the legs (UPDRS III item 26). Correlations were performed for each condition separately (StimOff, STN, and SNr’) and calculated with Spearman tests using SPSS 22.0. All tests were decided on a two-sided significance level of *p* < 0.05.

## Results

### Frequency Domain Analysis of Muscular Activity

#### Healthy Controls vs. Parkinson’s Disease in ‘Stimulation Off’

**FIGURE 1:**
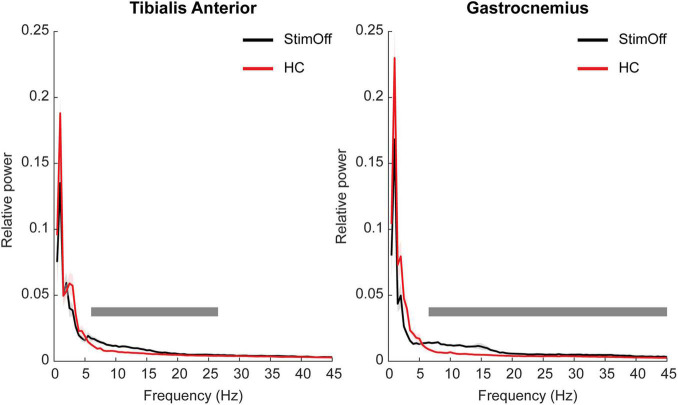
Power spectrum and standard error of the mean (SEM) of TA (**A,** left panel) and GA (**B,** right panel) during ‘regular gait’ in 9 PD patients with DBS turned off (‘StimOff’) and healthy controls (HC). PD patients in ‘StimOff’ showed higher power of TA in the alpha and low-beta range (6–26.5 Hz; *p* = 0.0004) and a higher power in the GA muscle (6.5–45 Hz; *p* = 0.0004, cluster based permutation test).

Parkinson’s disease patients in ‘StimOff’ showed higher power of both the TA and the GA compared to HC in the cluster-based statistical comparison from 1 to 45 Hz during ‘regular gait.’ In TA, this was represented in the alpha and low-beta range (6–26.5 Hz; *p* = 0.0004). In GA, it covered a broad frequency range from 6.5 to 45 Hz (*p* = 0.0004) (Figure 1).

To exclude that the low-frequency cut-off filter < 10 Hz affected our findings, we added a control analysis, i.e., we re-calculated the muscular frequency domain spectra after bandpass filtering from 1 to 200 Hz (refer Supplementary Figure 1). Statistical analysis revealed similar results: PD patients in ‘StimOff’ showed higher power of both the TA and the GA compared to HC in the cluster-based statistical comparison from 1 to 45 Hz during ‘regular gait.’ In TA, this was represented in the alpha and low-beta range (5.5–26 Hz; *p* = 0.0004), in GA from 6 to 45 Hz (*p* = 0.0004).

We asked next, whether the increased activity increase of the alpha and beta band observed in PD freezers in ‘StimOff’ was related to FoG or to the PD motor ‘off state’ more generally. To this end, we studied the spectra in ‘StimOff’ and compared first the frequency domain spectra between ‘regular gait’ vs. ‘freezing’ episodes. Then, we studied whether neurostimulation of STN or SNr modulated the muscular low-frequency activity.

#### ‘Regular Gait’ vs. ‘Freezing Episodes’ in ‘Stimulation Off’

**FIGURE 2:**
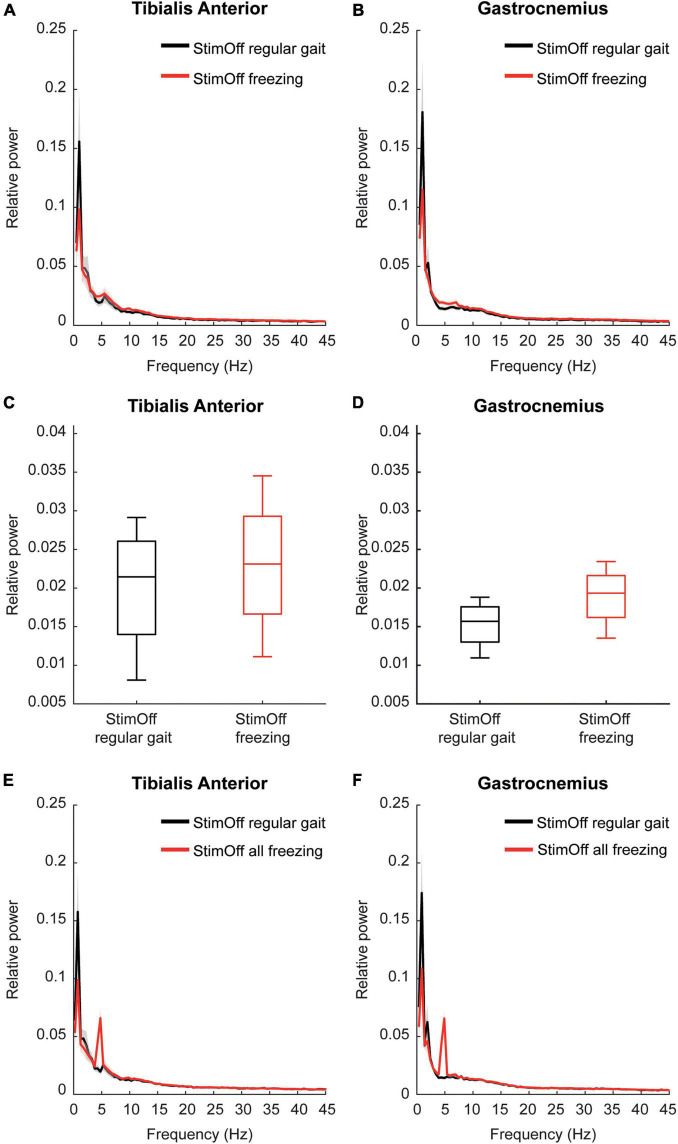
**(A,B)** Power spectrum and SEM of TA (upper left panel) and GA (upper right panel) in 4 PD patients with ‘StimOff’ comparing ‘regular gait’ and ‘freezing.’ Freezing episodes showed higher median power in TA and GA between 5 and 10 Hz, however, this difference did not show significance and has to be considered with caution owing to the limited number of patients showing freezing episodes during straight walking. **(C,D)** Alpha/low-beta power observed in ‘StimOff‘ during regular gait and freezing episodes in TA (middle left panel) and GA (middle right panel). There was no significant difference between the two conditions. Data is presented in boxplots giving the median, 25, 75th percentile, min and max. **(E,F)** Power spectrum and SEM of TA (lower left panel) and GA (lower right panel) in 5 PD patients with ‘StimOff’ comparing ‘regular gait’ and ‘ all freezing’ episodes. Freezing episodes showed higher median power in TA and GA between 5 and 10 Hz, however, this difference did not show significance and has to be considered with caution owing to the limited number of patients showing freezing episodes. Please note the prominent peak at 5 Hz in TA and GA. Visual inspection of the video recordings as well as of the EMG raw data did not reveal tremor-associated activation. Further, the individual frequency spectra of the 5 subjects did not show similar peak activation but a broader low-frequency activation band known from freezing episodes (Moore et al., 2008; Vercruysse et al., 2012) as opposed to tremor that occurs in a stable circumscribed frequency bin in the individual subject.

The frequency domain spectra pointed to higher activity in both TA and GA during ‘freezing’ episodes compared to ‘regular gait’ in 4 PD freezers, however, this did not reach statistical significance in the cluster-based comparison of the frequency domain spectra from 1 to 45 Hz (TA: *p* = 0.1264; GA: *p* = 0.1204) (Figures 2A,B). When specifically comparing the individual peak maxima of the alpha/low beta frequency range as our main frequency range of interest (6–26.5 Hz as derived from our previous analysis PD StimOff vs. HC), we found higher peak maxima in ‘freezing’ compared to ‘regular gait,’ however, again this did not reach statistical significance (TA: *p* = 0.250; GA: *p* = 0.375, Wilcoxon signed rank test) (Figures 2C,D).

In further subanalysis we compared ‘all freezing episodes’, regardless of whether they occurred during regular gait or during a U-turn in ‘StimOff’ and ‘regular gait’. This accounted for 24 freezing episodes in 5 patients and an absolute time frozen of 266 s. Again, the frequency domain spectra pointed to higher activity in both TA and GA during ‘all freezing episodes’ compared to ‘regular gait,’ however, this did not reach statistical significance in the cluster-based comparison of the frequency domain spectra from 1 to 45 Hz (TA: *p* = 0.7030; GA: *p* = 0.2244) (Figures 2E,F).

#### ‘Subthalamic Nucleus’ or ‘Substantia Nigra Pars Reticulate’ vs. ‘Stimulation Off’

**FIGURE 3:**
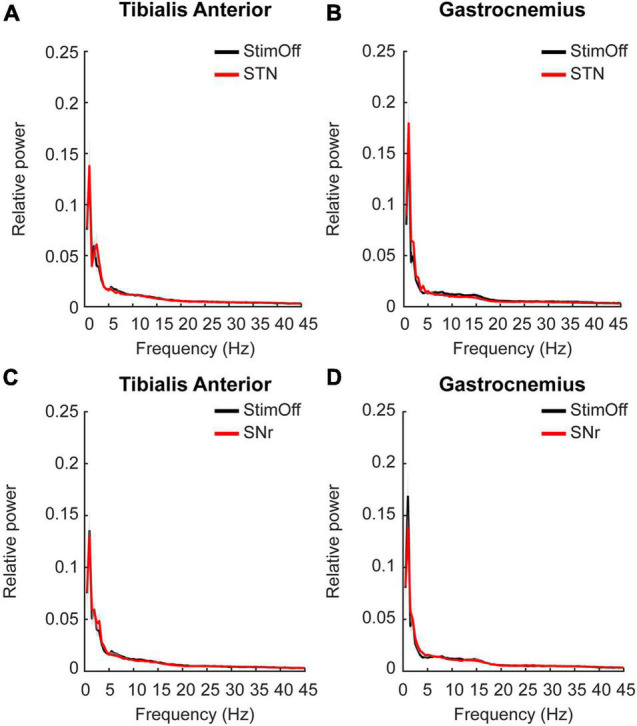
**(A,B)** Power spectrum and SEM of TA (upper left panel) and GA (upper right panel) during ‘regular gait’ in PD patients with ‘StimOff’ vs. ‘STN’. There was no significant difference in the power spectra of the two conditions. **(C,D)** Power spectrum of TA (lower left panel) and GA (lower right panel) during ‘regular gait’ in PD patients with ‘StimOff’ vs. ‘SNr.’ There was no significant difference in the power spectra of the two conditions.

Next, we studied whether muscular activity during ‘regular gait’ in ‘StimOff’ was modulated by either ‘STN’ or ‘SNr’ stimulation. STN showed lower activity in TA between 5 and 21 Hz and in GA from 7 to 23 Hz, however this did not reach statistical significance in the cluster-based comparison from 1 to 45 Hz (‘StimOff’ vs. ‘STN’: TA: *p* = 0.6111, GA: *p* = 0.8362; ‘StimOff’ vs. ‘SNr’: TA: *p* = 0.3639, GA: *p* = 0.1784) (Figures 3A–D).

**FIGURE 4:**
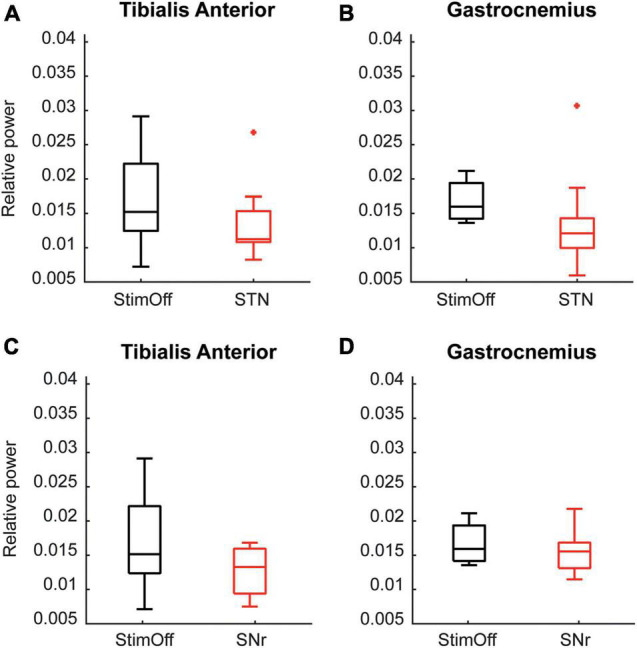
**(A,B)** Data is presented in boxplots giving the median, 25th, 75th percentile, min and max. Alpha/low beta power observed in ‘StimOff’ can be significantly lowered by ‘STN’ in both the TA (upper left panel, *p* = 0.015) and GA (upper right panel, *p* = 0.015). **(C,D)** Data is presented in boxplots giving the median, 25th, 75th percentile, min and max. There was no significant difference of the alpha/low beta power observed in ‘StimOff’ vs. ‘SNr’ in both the TA (lower left panel) and GA (lower right panel). ^+^Indicates statistically significat differences.

As derived from the contrast PD StimOff vs. HC, we further analyzed as additional non-parametric analysis the individual peak maxima from 6 to 26.5 Hz. We found that ‘STN’ (TA: *p* = 0.015, GA: *p* = 0.015, Wilcoxon signed rank test), but not ‘SNr’ (TA: *p* = 0.093, GA: *p* = 0.161) decreased the peak maxima in the alpha/low-beta range in both TA and GA compared to ‘StimOff’ (Figures 4A–D).

#### Correlations of Alpha/Low-Beta Peak Maxima With Clinical Motor Scores and Freezing of Gait Measures

**FIGURE 5:**
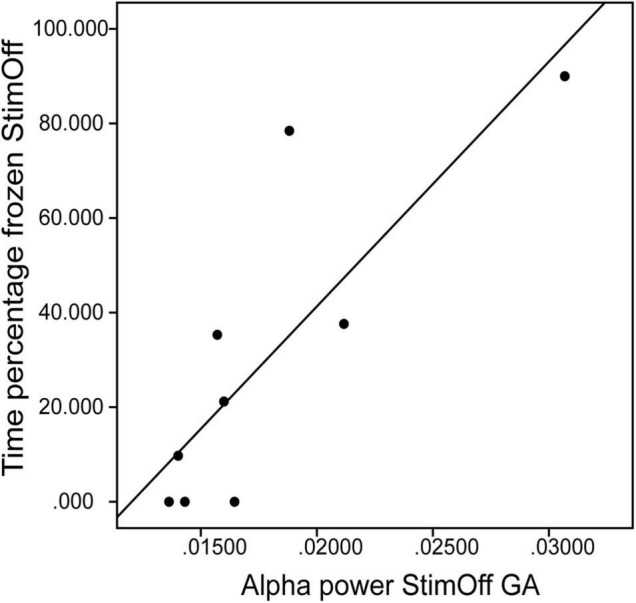
Correlation between the alpha/low beta peak maxima of the GA (*x*-axis) and the time percentage frozen in ‘StimOff’ (*r* = 0.763, *p* = 0.017). Higher alpha/low beta peak maxima are associated with longer freezing duration.

We correlated the individual alpha/low beta peak maxima in ‘StimOff’ with clinical measures of FoG and control variables. As freezing measures there was a correlation of time percentage frozen and the alpha/low-beta peak maxima of the GA in ‘StimOff’ (*r* = 0.763, *p* = 0.017, uncorrected; Figure 5) but not of the TA (*p* = 0.631). There was no correlation of NFOG-Q (TA: *p* = 0.439; GA: *p* = 0.841), UPDRS III freezing (item 14) (TA: *p* = 0.489, GA: *p* = 0.768), and the number of FoG episodes (TA: *p* = 0.452, GA: *p* = 0.965). As control analyses, we did not find correlations with rigidity (UPDRS III item 22) (TA: *p* = 0.795, GA: *p* = 0.931) and diadochokinesia (UPDRS III item 26) (TA: *p* = 0.628, GA: *p* = 0.742).

### Gait Characteristics

On average, HC walked for 198 ± 55 s (mean ± standard deviation) with a cadence of 105 ± 10 steps per minute and a step length of 0.46 ± 0.05m. PD patients in ‘StimOff’ walked on average 126 ± 53 s with a cadence of 106 ± 16 steps per minute and a step length of 0.23 ± 0.09 m. In ‘STN,’ PD patients walked on average 157 ± 36 s with a cadence of 101 ± 15 steps per minute and a step length of 0.28 ± 0.08 m. In ‘SNr,’ PD patients walked 151 ± 57 s, with a cadence of 108 ± 18 steps per minute, and a step length of 0.21 ± 0.08 m.

We performed a statistical comparison that revealed significant differences in stride length between HC and PD in ‘StimOff’ (*p* = 0.00006, Mann–Whitney-*U*-Test). Since gait speed is known to have an impact on additional kinematic parameters (Fukuchi et al., 2019), we additionally calculated the over ground walking speed in HC: 0.7 ± 0.1 m/s, and in PD in ‘StimOff’: 0.4 ± 0.1 m/s (*p* = 0.00007, Mann–Whitney-*U*-Test), which also differed significantly between the two groups. These kinematic differences might also affect underlying electrophysiological differences between HC and PD in ‘StimOff.’ Therefore, we correlated individual gait speed to (i) the frequency of the individual alpha peak (ρ = –0.51, *p* = 0.16) and (ii) to the relative power of the individual alpha peak (both muscles: ρ = –0.42, *p* = 0.26; TA ρ = –0.07, *p* = 0.87; GA ρ: –0.45, *p* = 0.13). All correlations were negative and do not suggest that lower EMG power relates to gait speed.

## Discussion

In this study, we studied PD freezers while walking with ambulatory EMG-recordings of the antagonistic leg muscles. We found that PD freezers showed enhanced activity of TA and GA in a low frequency range of alpha and beta band, presumably in both TA and GA compared to HC during regular walking. Furthermore PD freezers showed a similar activation profile during regular walking and actual freezes. Interestingly, STN stimulation decreased this pathological activity together with improved clinical outcomes in freezing.

### Pathological Muscular Activity and Its Relation to Freezing in Parkinson’s Disease

In healthy people EMG activity of hand movement and fine motor task is mostly located in the beta band (15–30 Hz) or Piper rhythm (35–60 Hz) (Brown, 2000), in contrast to PD patients, who showed predominant activation around or below 10 Hz in EMG in dopaminergic off state. Effective L-Dopa or STN-DBS therapy lead to suppression of this pathological activity (Salenius et al., 2002; Weiss et al., 2012). However, the relation of these activation abnormalities to PD gait remained unknown. We found that PD freezers show elevated activity from 6 to 45 Hz in the antagonistic leg muscles during effective regular gait, and this was centered in the low frequency range of the alpha and low beta bands. This was present during both regular gait and during the freezes to similar extent. Since the activity was not specific to the freeze itself but existed already during preserved gait, muscular activation at lower frequency around and below 10 Hz may be a general feature of the PD off state mirroring pathological supraspinal activation patterns on the level of the spinal cord (Salenius et al., 2002; Weiss et al., 2012; Flood et al., 2019).

Nevertheless, the finding raises considerations on whether activation at lower frequency contributes to freezing, such that it could represent a more unstable motor system which yields susceptibility to encounter freezing episodes (Scholten et al., 2016b). Activation abnormalities around and below 10 Hz were identified as a pathological feature of the multistage locomotor network comprising cortex (Marsden et al., 2001; Scholten et al., 2016a), basal ganglia (Hammond et al., 2007; de Solages et al., 2010; Chen et al., 2019), brainstem (Thevathasan et al., 2012), and spinal pattern generator (Marsden et al., 2001; Weiss et al., 2012; Scholten et al., 2016a). From these data and the present study it is plausible to reason that these activation abnormalities relate to freezing, since freezers show premonitory activation abnormalities already outside or immediately preceding a freeze in contrast to non-freezers (Singh et al., 2013; Toledo et al., 2014; Storzer et al., 2017). Instability of the motor system may be a pre-requisite to freezing there mirroring the susceptibility of a PD patient to loose effective spinal motor output (Scholten et al., 2016b,^2020^). During a freeze itself, the pathological rhythm can either stay unchanged or even increase (Scholten et al., 2016a; Anidi et al., 2018; Chen et al., 2019; Pozzi et al., 2019).

In this sense, we speculate that low-frequency activation of the spinal pattern generator of PD freezers could be interpreted as an abnormally slow and prominent rhythm. This might oppose the rapid adjustment of antagonistic muscle activity as is needed for the recurrent cycling of activation and deactivation cascades throughout the cyclic gait phases. Mathematically spoken, motor output in the high beta and gamma range > 20 Hz would allow for much faster reprogramming and adaptation of the spinal pattern generator (Schoffelen et al., 2005) as opposed to the activation abnormalities at slower rhythms observed in this freezer group. This would lead – depending on the individual cadence – to a much slower adaptation and also performance of the alternating activation – deactivation changes of the antagonistic leg muscles throughout the full gait cycle, which can only interact in the slow alpha and low-beta rhythms in PD. In healthy subjects activation of the TA will take for about 10% of the gait cycle, from initiation to full recruitment (Hart et al., 2006). Considering a cadence of around 105 steps per minute in our cohort of healthy controls indicates a duration of the gait cycle of 0.57 s and a necessity of adaptions within 57 ms. The prominent slow alpha and low-beta rhythms in PD patients will not allow for more than a temporal resolution of around 50–100 ms, which is inherently slow to allow rapid adjustments of the gait cycles and leads to abnormal temporal activation patterns in PD freezers (Nieuwboer et al., 2004). Moreover, the ability to adapt gait to the external or internal requirements is limited at such slow frequencies, and this may comply with the clinical observation that gait performance can be disrupted with cognitive interference (Nutt et al., 2011; Nonnekes et al., 2015). Instead, it was stabilized with rhythmic cueing, as was the oscillatory beta activity time-locked to the gait cycle (Fischer et al., 2018).

Besides the enhanced alpha and low beta activity our data also reveal higher power spectra > 20 Hz in GA, which could be due to (i) either postural differences in PD patients, caused by a more flexed posture (Nieuwboer et al., 2004) and a tendency to shift the center of gravity forward when walking, resulting in toe walking or (ii) reflect compensatory mechanisms caused by cortical drives to the spinal cord which are located in a higher frequency range (Schoffelen et al., 2005). To this point it is rather difficult to disentangle whether this high frequency activation in GA is a causal or compensatory mechanism in the first place.

### Subthalamic Stimulation Reduces Muscle Activation at Low Frequencies and Clinical Freezing Outcomes

Subthalamic nucleus -stimulation in PD is a potent treatment for L-Dopa sensitive FoG. Clinical studies reported on reduced occurrence of FOG and sever falls in PD freezers after undergoing STN-DBS (Schlenstedt et al., 2017; Barbe et al., 2020; Cebi et al., 2020). However, to prevent adverse outcomes of STN-DBS it is important to avoid co stimulation of the pallido-thalamic tract crossing on the level of the zona incerta as was for example found in antero-medially displaced electrodes or by delivering large amounts of energy and increasing the electrical field (Moreau et al., 2008; Fleury et al., 2016). When applying effective STN-stimulation along with clinical improvement also pathological changes of gait parameters in PD freezers, such as stride length and stride amplitude improve (Pötter-Nerger and Volkmann, 2013; Scholten et al., 2017). Notably, high frequency stimulation of the STN also led to a reduction of beta-band activity in STN-LFPs. In our experiment the kinematic parameters do not reveal significant differences between ‘STN’ and ‘StimOff.’ Scholten et al. (2017) report results of a larger group of PD freezers in which gait parameters, temporal and spatial, showed improvement with STN- or SNr-stimulation. Especially the stride length improved when applying STN-DBS. In the subgroup we choose for our data analysis also the stride length improves in STN-condition, but the changes are not statistically significant, probably due to the smaller size of the cohort. Additionally also the absolute number of freezing episodes across subjects and the absolute time frozen improved when we applied STN-DBS in contrast to the ‘StimOff’-condition, and this clinical improvement was associated with a reduction of pathological activity at low frequency in TA and GA. The modulation effect on oscillatory patterns of STN-DBS is not locally limited to the STN, but may impact on the functionally connected areas, in particular the subthalamo-cortical circuits (de Hemptinne et al., 2015; Weiss et al., 2015). On the one hand STN has an excitatory net effect on SNr and GPi, resulting in more inhibitory control on PPN/MLR which was brought in context to FoG (Shine et al., 2013; Weiss et al., 2019). STN-DBS is attenuating the exaggerated glutamatergic output (Benabid et al., 2003) and the pathological beta oscillations (Kühn et al., 2008; Eusebio et al., 2012). This means that STN-DBS could potentially act on releasing the pallidothalamic inhibition of the primary motor cortex, or by modulating the cortex more directly via the hyperdirect connections. However, toning down pathological STN activity could also change SNr activity through the monosynaptic connections (Milosevic et al., 2018; Weiss et al., 2019). Thus, observing a change of muscular activity with STN-DBS would not necessarily mean that the effect is transmitted via the ‘ascending’ cortical and then corticospinal pathway, but would still allow for contributions of the ‘descending’ nigropontine route. However, our second finding that SNr_mono_ stimulation did not affect both muscular activity and clinical FoG measures argues against this alternative interpretation. Therefore, we propose that the primary effect of high-frequency STN-DBS was delivered via the subthalamo-cortical circuits of either the indirect or the hyperdirect pathway – or maybe both (Gradinaru et al., 2009; de Hemptinne et al., 2015). STN holds projections via the subthalamo-pallido-cortical ‘hyperdirect’ and the striato-external pallido-subthalamo-internal pallido-cortical ‘indirect’ pathway, both executing inhibitory control on thalamo-cortical activation patterns and preventing effective motor output (Delwaide et al., 2000). STN-DBS can effectively modulate the oscillatory patterns of cortical areas via these pathways and electrophysiological changes are accompanied by improved clinical outcome parameters (Kuriakose et al., 2010; de Hemptinne et al., 2015; Weiss et al., 2015). Considering the fact that in our experiment clinical as well as oscillatory improvements were achieved via STN-DBS, but not SNr-DBS, it seems reasonable to consider the underlying pathological enhance alpha and low beta activity also being primary transmitted via the subthalamo-cortical pathway and the descending projections of the pyramidal tract to spinal motor neurons. Further support for this hypothesis comes from studies on the pathology of ULF, when freezing episodes were associated with increased cortical activity (7–11 Hz), muscular activity (6–9 Hz), and increased intermuscular coherence, the latter of which was proposed as marker for cortical control of muscular activity (Scholten et al., 2016a). Nevertheless, cortical contributions do not exclude subcortical contributions to muscular activation at low frequencies. Future research using combined EMG-LFP-EEG measurement in gait paradigms will help to further investigate the underlying network interactions of pathological activation pattern in FoG (Kühn et al., 2009; Lewis and Barker, 2009; Anidi et al., 2018; Pozzi et al., 2019). Especially high resolution time-frequency analyses of LFP-EEG data could help differentiate a primary cortical or subcortical source of the muscular activation abnormalities of the alpha and beta band.

### Methodological Considerations

In this study, we investigated the role of muscular activation abnormalities at alpha and beta frequencies for FoG taking advantage from ambulatory EMG recordings, and studied their supraspinal modulation with STN- and SNr-DBS. We were able to analyze data from a very homogenous group of finally 9 PD freezers with an acinetic-rigid subtype, susceptibility of FoG and electrode localization of the most caudal contact being located in SNr, a group size comparable to those of previous electrophysiological studies (Anidi et al., 2018; Pozzi et al., 2019). Although observing a large quantity of freezes under laboratory conditions has been recognized as a challenge in FoG research (Lewis and Shine, 2016; Weiss et al., 2019) we were able to gather an absolute time of 144 s frozen in ‘StimOff,’ which can be considered sufficient data material to stabilize the frequency domain spectra from a statistical viewpoint (Mima and Hallett, 1999). A limitation is that the data of freezing episodes stem from only 5 of the 9 subjects which limits the interpretation and generalizability of comparing the spectra of the freezing state with regular gait. The same applies for comparisons of clinical features, i.e., number of freezing episodes, absolute time frozen, etc. The descriptive indicate a marked reduction of freezing, considering the number of patients expressing FoG (4 in ‘StimOff,’ 1 in ‘STN’), the number of freezing episodes (15 in ‘StimOff,’ 2 in ‘STN’) and the absolute time frozen (144 s in ‘StimOff,’ 50 s in ‘STN’). Still the subgroup is quite small and performing a Wilcoxon signed rank test would not show statistical significant improvement. But we were therefore careful in interpreting our finding, and suggest to leave it to future studies to test, whether muscular activity shows a further increase during freezes as compared to regular gait. Nevertheless, the above summarized evidence across the distributed freezing-network levels draws a coherent picture that activation around and below 10 Hz in basal ganglia, brainstem, cortex, and spinal motor neurons is critical to freezing susceptibility, i.e., representing a general failure of neuronal gait integration in PD freezers that yield a risk for expressing freezing episodes on this grounds.

We performed several correlation analyses of clinical measurements and electrophysiological parameters. There was no correction for multiple comparisons, since we performed the correlation analysis with exploratory intent and did not interpret them in a confirmatory way. Instead, we suggest to reproduce these findings in independent and larger cohorts.

## Conclusion

Here, we demonstrated with ambulatory EMG that PD freezers in medication off and stimulation off show abnormal activation of alpha and low-beta band activity when compared with healthy subjects. This was not specific to the freezing state. However, our findings and the context to the available research support that activation abnormality contributes to freezing susceptibility, since STN-DBS decreased the muscular activity together with clinical improvement of FoG. Since we found that STN-DBS but not SNr-DBS was effective to suppress this low-frequency activity, it is likely that the cortical projections of the STN – rather than the brainstem connections albeit not being exclusive – were meaningful to this effect. Future combined LFP-EEG-EMG research may shed further light on the neuronal supraspinal contributions to muscular activation abnormalities.

## Data Availability Statement

The data analyzed in this study is subject to the following licenses/restrictions: privacy policy of individual patients’ data. Requests to access these datasets should be directed to DW, daniel.weiss@med.uni-tuebingen.de.

## Ethics Statement

The studies involving human participants were reviewed and approved by Ethik-Kommission am Universitätsklinikum Tübingen. The patients/participants provided their written informed consent to participate in this study.

## Author Contributions

MS, AG, and DW: conception and design of the study. MS and JK: acquisition of data. IC: patient inclusion. MS, M-SB, and DW: analysis and interpretation of data. M-SB and DW: drafting the manuscript. All authors: critical revision and final approval of the version to be submitted.

## Conflict of Interest

DW received honoraria and/or research support from Abbot, Abbvie, Bial, Boston Scientific, Kyowa Kirin, Medtronic, and Stada pharma. The remaining authors declare that the research was conducted in the absence of any commercial or financial relationships that could be construed as a potential conflict of interest.

## Publisher’s Note

All claims expressed in this article are solely those of the authors and do not necessarily represent those of their affiliated organizations, or those of the publisher, the editors and the reviewers. Any product that may be evaluated in this article, or claim that may be made by its manufacturer, is not guaranteed or endorsed by the publisher.

## Supplementary Material

Supplementary Figure 1 Power spectrum and standard error of the mean (SEM) of TA **(left panel)** and GA **(right panel)** during ‘regular gait’ in 9 PD patients with DBS turned off (‘StimOff’) and healthy controls (HC) after band pass filtering of 1–200 Hz. PD patients in ‘StimOff‘ showed higher power of TA in the alpha and low-beta range (5.5–26 Hz; *p* = 0.0004) and a higher power in the GA muscle (6–45 Hz; *p* = 0.0004, cluster based permutation test).

## Abbreviations

DBS: deep brain stimulation
PD: Parkinson’s disease
HC: healthy controls
STN: subthalamic nucleus
SNr: substantia nigra pars reticulate
MLR: mesencephalic locomotor region
PMRF: ponto-medullary reticular formation
EEG: electroencephalography
EMG: emelctromyography
ULF: upper limb freezing
TA: tibialis anterior
GA: gastrocnemius
PPN: pedunculo-pontine nucleus
TO: toe-off
HS: heel-strike
MS: midswing
FFT: fast Fourier transform.
