# Supplementary figures and images for: People With Parkinson’s Disease and Freezing of Gait Show Abnormal Low Frequency Activity of Antagonistic Leg Muscles
Source: Front Hum Neurosci. 2022 Jan 26;15:733067. doi: 10.3389/fnhum.2021.733067 (PMC8825470; doi:10.3389/fnhum.2021.733067)

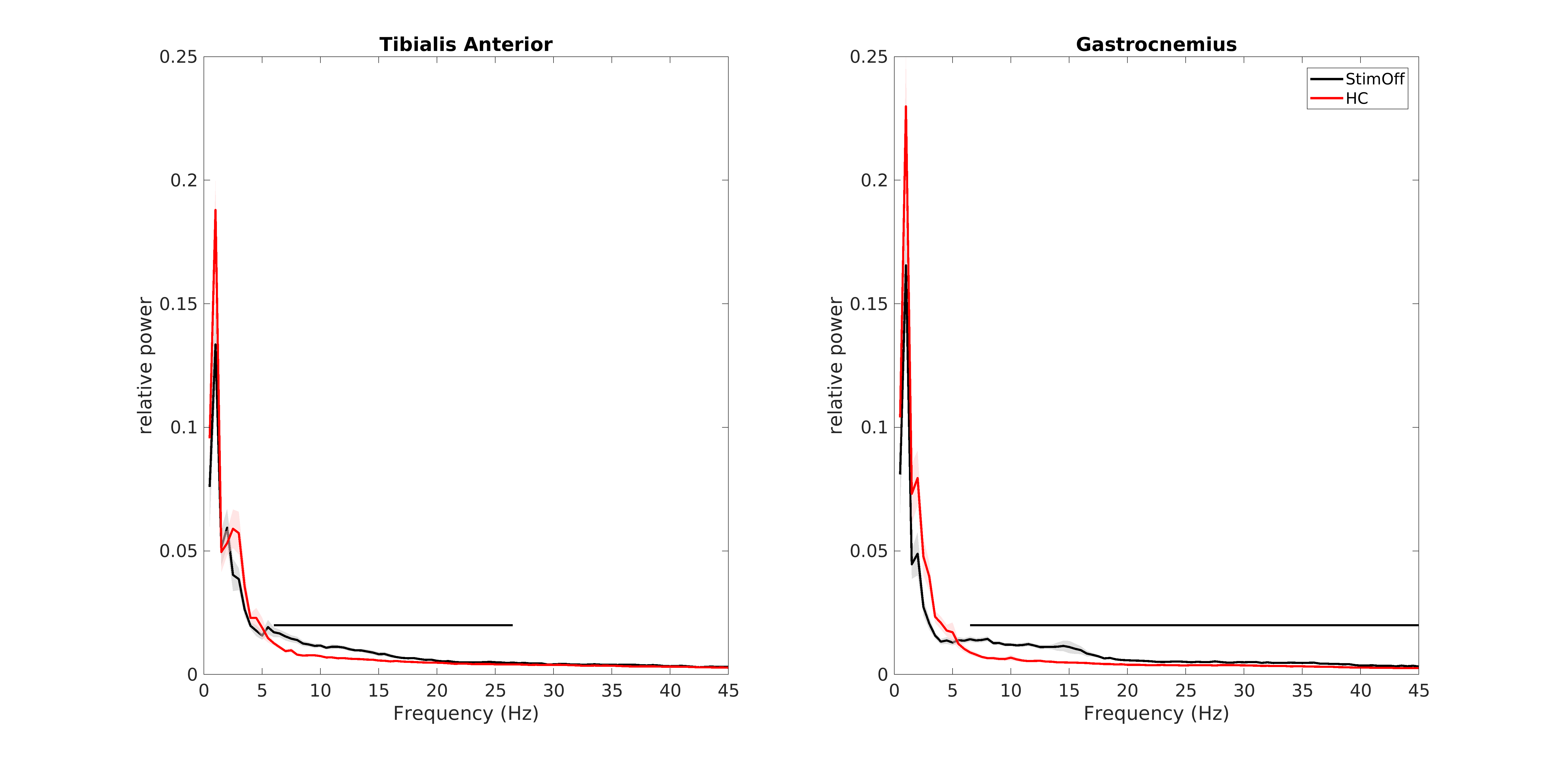

Supplement: Supplementary Figure 1 — Power spectrum and standard error of the mean (SEM) of TA (left panel) and GA (right panel) during ‘regular gait’ in 9 PD patients with DBS turned off (‘StimOff’) and healthy controls (HC) after band pass filtering of 1–200 Hz. PD patients in ‘StimOff‘ showed higher power of TA in the alpha and low-beta range (5.5–26 Hz; p = 0.0004) and a higher power in the GA muscle (6–45 Hz; p = 0.0004, cluster based permutation test). [file Image_1.tif]
